# Investigation of the effects of balance exercises on visuospatial skills using EEG brain oscillations

**DOI:** 10.1007/s11571-026-10494-4

**Published:** 2026-06-26

**Authors:** Yasin Yıldırım, İrem Yemeniciler, Devrim Tarakcı, Bahar Güntekin

**Affiliations:** 1 Faculty of Health Sciences, Department of Physiotherapy and Rehabilitation, Istanbul Gedik University, Istanbul, Turkey; 2 Department of Neuroscience, Institute of Health Sciences, Istanbul Medipol University, Istanbul, Turkey; 3 Faculty of Health Sciences, Department of Ergotherapy, Istanbul Medipol University, Istanbul, Turkey; 4 Brain and Cognition Research Center, BEYKOG, SABİTA, Istanbul Medipol University, Istanbul, Turkey; 5 Department of Physiotherapy and Rehabilitation, Institute of Health Sciences, Istanbul Medipol University, Istanbul, Turkey; 6 Department of Biophysics, School of Medicine, Istanbul Medipol University, Istanbul, Turkey

**Keywords:** Brain oscillations, EEG, Exercise, Mental rotation, Visuospatial attention

## Abstract

Visual-spatial attention (VSA) selects relevant sensory information and supports the preparation of responses to this information. Mental rotation (MR) is the ability to rotate an object seen from a certain perspective to a new orientation in space. Exercise stands out as a promising non-pharmacological treatment for cognitive functions. Balance control is known to be related to the visual system. Therefore, the aim was to investigate the effectiveness of video-based balance games and structured balance exercises on VSA and MR with EEG brain oscillations. 30 healthy participants were included in the study. Participants were divided into two groups (structured balance exercises group (SBEG) and video-based balance exercises group (VBBEG)) by randomization. Both groups received exercise sessions 2 days a week for a total of 6 weeks. The mentioned cognitive functions were evaluated by selecting tests previously used in the literature. For the VSA task, after 6 weeks of exercise, occipital theta (4–7 Hz) power decreased in the VBBEG group, while SBEG increased. In the MR task results, high alpha (11–13 Hz) power decreased in VBBEG and increased in SBEG when centroparietal areas were examined. In conclusion, it is thought that the two different exercise methods may affect visual-spatial attention and mental rotation skills in different ways.

**Supplementary Information:**

The online version contains supplementary material available at 10.1007/s11571-026-10494-4.

## Introduction

Visual-spatial skills are of great importance for functional independence; they enable us to establish 2- and 3-dimensional relationships with our environment, to perceive the shapes of objects in space, to understand the location of objects in space and the spatial orientation of our body (Marshall and Fink 2001; de Bruin et al. 2016). Visual-spatial skills (VSS) require the proper processing of information received from the vestibular, somatic, proprioceptive, visual, auditory systems and gravitational afferentation (Kozlovskaya et al. 1985). VSS is divided into components such as visuospatial attention (VSA), visuospatial memory, and mental rotation (MR) (Mountcastle 1995). VSA allows for the selection and suppression of incoming visual information (Gallotto et al. 2020). MR is defined as the ability to rotate an object viewed from a certain perspective to a new orientation in space and determine how it will appear when viewed from another perspective (Moore and Johnson 2020).

It is known that cognitive and motor skills develop in a coordinated manner both in children and in older ages and that there is an essential relationship between them (Frick and Möhring 2016). Embrechts et al. (2021) investigated the relationship between VSS and balance in stroke patients. Their study revealed that decreased VSS can cause balance and posture problems. It has been suggested that visuospatial input is essential for proactive planning and adjustments to maintain stability in dynamic and complex environments and allows for the preventive regulation of movement patterns that provide safe movement and postural control (Mak et al. 2021).

Exercise stands out as a promising non-pharmacological treatment for cognitive functions (Huang et al. 2022). A large amount of evidence from studies has shown that exercise is effective in protecting brain health and cognitive functions in both normal individuals and individuals with neurodegenerative diseases (Cassilhas et al. 2016). Although the exact mechanisms through which exercise affects the brain are not yet fully understood, neuroplasticity is considered a key underlying mechanism. A large body of research shows that neuroplasticity can be stimulated by acute or chronic exposure to physical exercise (Vints et al. 2022). When the treatment options for VSA in the literature are investigated, it is understood that a consensus has not yet been established. This may be because VSA is affected in many different variations and is seen together with various diseases. Darestani et al. (2020) concluded that an application called RehaCom, which includes cognitive training, may be beneficial for VSS in individuals with Multiple Sclerosis. Adomavičienė et al. (2019) found that both applications positively affected visuospatial skills in individuals with stroke in their studies using the upper extremity robot called Armeo Spring and Xbox Kinect systems. When the relationship between MR and exercise was investigated, a very rich content could not be found in the literature. Jansen et al. (2010) found that a 45-minute training consisting of running, jumping and calisthenic exercises was effective on MR. Many studies investigate the effectiveness of balance training in healthy individuals (Bateni 2012; Nicholson et al. 2015; Wu et al. 2023). Classical balance exercises and video-based game therapy stand out among the methods used during balance training. DiStefano et al. (2009) concluded in their published review that classical balance exercises improve balance in healthy adults. Chen et al. (2021) found that video-based games positively affected balance in healthy participants.

Neuroimaging and lesion studies have demonstrated the involvement of a widespread functional network in the control of VSA, including centers in the frontal, parietal, temporal, and occipital cortices (Wiesman et al. 2017). When the literature is searched, it is seen that theta (4–7 Hz) and alpha (8–13 Hz) oscillations related to visual-spatial attention have been studied the most (Händel et al. 2011). Rhythmic behavioral patterns, especially neurons that serve the active exploration of the environment (e.g., eye movements) or communication (e.g., speech production), are typically activated at frequencies in the theta range, i.e., around 3–8 Hz (Benedetto and Morrone 2019). It has been reported that theta oscillations can encode multiple elements in a defined order or spatial organization and regulate and control information flow between distant regions (Rawle et al. 2012). Bengson et al. (2014) asked subjects to direct their attention to a specific direction and found an increase in potential in the frontal regions and parietal area between 250 and 350 milliseconds (ms) and 400–800 ms. During the MR task, structures such as the frontal region, premotor area, occipital region, superior parietal cortex, inferior temporal cortex, supplementary motor area play an active role (Nishimura et al. 2020). The literature has focused on changes in alpha power in MR. The decrease in mu (8–13 Hz) power over the sensorimotor cortex is thought to be due to the underlying motor rotation during the task (Gardony et al. 2017). It has been reported that the decrease in alpha activity in this region (due to its association with inhibition) is due to the creation of representations related to MR and the need for demand (De Lange et al. 2005). It has been stated that a high alpha (HA) (11–13 Hz) focus provides a higher spatial focus (Gardony et al. 2017). It has also been shown that changes in HA (11–13 Hz) power provide a valid basis for revealing the relationship between the involvement of visual and motor processes and the task (De Lange et al. 2005; van der Helden et al. 2010). In our study, we also preferred the 11–13 Hz range for alpha analysis. Horst et al. (2013), in their study evaluating MR through hand images, found decreases in HA (11–13 Hz) power during the task in the central, centroparietal and occipital areas. However, no study has been found examining the effect of balance exercises on VSA and MR using the EEG brain oscillations method. Therefore, this study aimed to investigate the effects of two different balance exercise methods on VSA and MR.

## Methods

### Participants

40 volunteers who met the inclusion criteria between the ages of 18–35 were included in the study. The study was conducted in the Cognitive Neuroscience and EEG, Technotherapy, Physiotherapy and Rehabilitation laboratories of Istanbul Medipol University. The study was conducted in accordance with the Declaration of Helsinki, approval for the study was obtained at the meeting of the Istanbul Medipol University Non-Interventional Clinical Research Ethics Committee dated 09.11.2023 with the file number E-10840098-772.02-7152. The participants included in the study were informed about the purpose, duration, treatments to be applied and the process of the study, and the “Written Informed Consent” was signed in accordance with the standards determined by the Istanbul Medipol University Clinical Research Ethics Committee, and their approvals for the study were obtained. The trial was registered at ClinicalTrials.gov (NCT06151093).

Inclusion criteria were age 18–35, right-handedness, no balance-affecting systemic conditions (e.g., vertigo or musculoskeletal disorders), no prior balance training, no neuropsychiatric diagnosis or medication use, and no history of foot–ankle surgery. Exclusion criteria included a history of orthopedic or neurological disease, epilepsy, musculoskeletal disorders, active use of virtual reality game consoles (e.g., Nintendo Wii, Xbox Kinect), regular exercise (e.g., gym, yoga, pilates), and color blindness.

### Randomization

Randomization was performed using a list-based system via the website www.randomizer.org.

### Experimental procedure

**Fig. 1:**
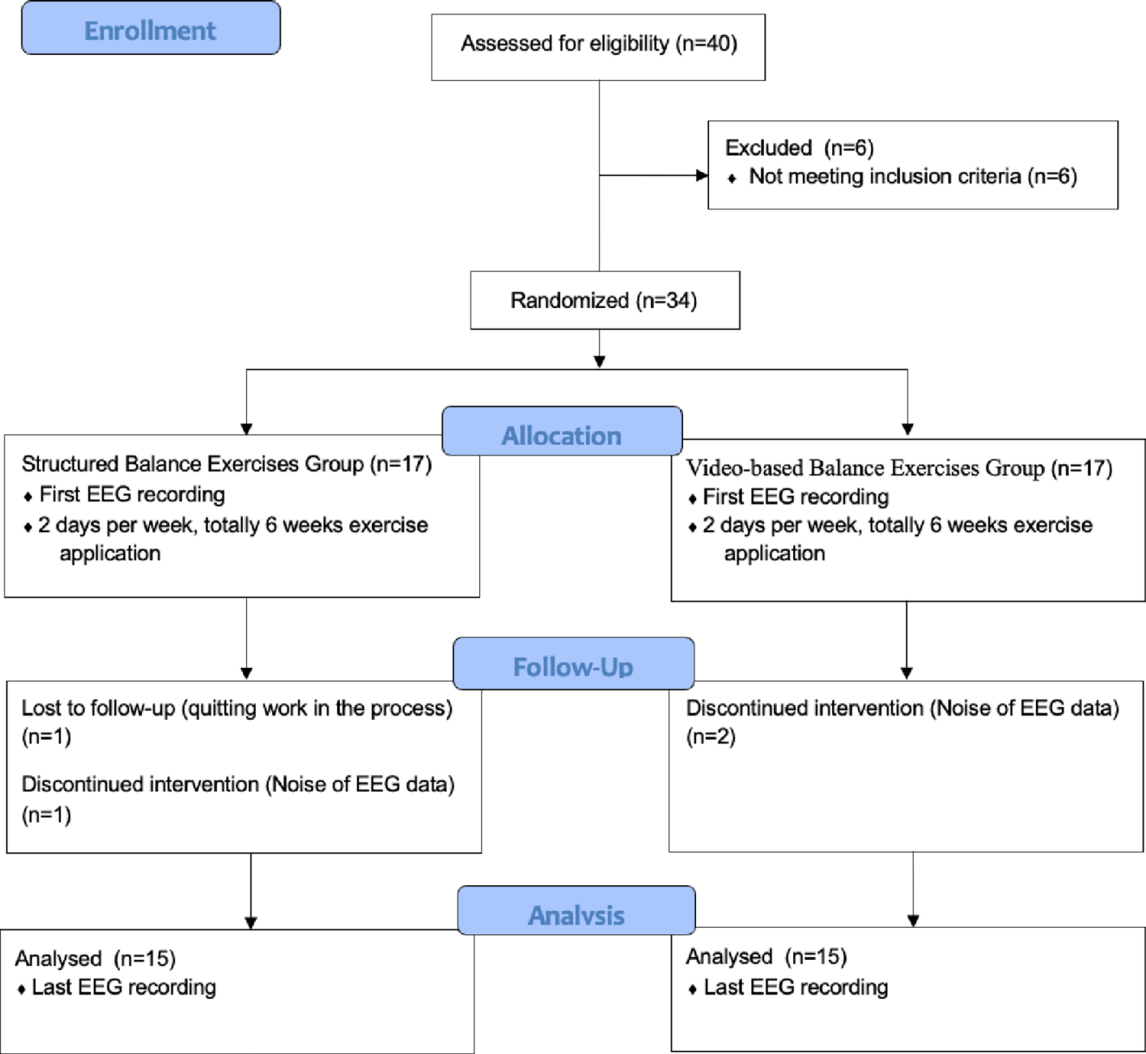
Flow chart

40 healthy individuals were evaluated for suitability for the study. Six people who did not meet the inclusion criteria were excluded from the study. Thirty-four participants who met the study criteria were included in the study group after they were informed and signed the consent form. The individuals were divided into two groups according to the randomization. The two groups in the study were the SBEG and the VBBEG. After the first EEG recording, the individuals were given exercise applications twice a week for a total of 6 weeks. The final EEG recordings were taken from the participants who completed the exercise applications (Fig. 1). Female participants were first assessed 14 days after the postmenstrual phase. This ensured that both pre- and post-assessments coincided with the ovulatory phase. The statements of female participants regarding menstruation reporting were accepted.

### Exercise intervention

Balance exercises to be performed in both virtual and real environments were planned for a total of 6 weeks. The exercises were applied on two different days per week, for a total of 12 sessions. Structured balance exercises were applied in Istanbul Medipol University Physiotherapy and Rehabilitation Laboratories, and video-based balance exercises were applied in Istanbul Medipol University Technotherapy Laboratory.

#### Structured balance exercises group

The exercises were composed of balance exercises; most used in physiotherapy and rehabilitation. Since the application was to be performed on healthy participants, a progressively more difficult process was followed. Each exercise was performed three times, both with eyes open and with eyes closed. All exercises were performed in front of a mirror to increase visual feedback. Each session lasted approximately 30–40 min. The exercise programs applied by week are given in Supplementary material (Suppl.).

#### Video-based balance exercises group

Video-based balance exercises were applied using Nintendo Wii. Nintendo Wii Fit, which is used in virtual reality treatment, is designed to increase aerobic capacity, grip, muscle strength, balance and upper extremity functionality. The game console consists of a motion-sensitive sensor, Wii remote (control that provides activity control), balance board and screen; and these mechanisms can be controlled wirelessly. The motion-sensitive sensor in Nintendo Wii Fit reflects the person’s movements on the screen thanks to the accelerometer which consists of three axes. The feedback on the screen provides self-control. Activities can be adapted to the personalized exercise program (Williams et al. 2011; Pompeu et al. 2012). The games and program details selected for Nintendo Wii video-based balance exercises are given below. Each game was applied three times in each session. A session lasted approximately 30–40 min. The games applied in the weekly flow, used in our study and their features are shown in Supplementary material (Suppl.).

### EEG recording

EEG recordings were performed with Brain Vision Recorder (Brainproducts, Munich, Germany). Recordings were made from 30 channels with 0.1–250 Hz band pass and 500 sample rate features. An elastic cap with 32 Ag-AgCl electrode placement was used as the cap. The placement of the electrodes was made according to the international 10–20 system and recordings were taken from FP1, FP2, F7, F3, FZ, F4, F8, FT7, FC3, FCZ, FC4, FT8, T7, C3, CZ, C4, T8, TP7, CP3, CPZ, CP4, TP8, P7, P3, PZ, P4, P8, O1, OZ and O2 electrodes. In addition, two electrodes (A1 + A2) connected to the anterior part of both earlobes were placed as reference, and the ground electrode was placed on the posterior part of the right earlobe. In addition to these, electrodes placed on the medial upper part of the left eye and the lateral orbital part were also used for EOG recording. Care was taken to ensure that the impedance values of the electrodes were below 10 kΩ during the recordings. EEG was amplified with a 0.01–250 Hz band range via a BrainAmp MR plus 32-channel DC system device (Brain Product GmbH, Germany). The online sampling rate was digitalized as 500 Hz. EEG recordings were performed using a dimly lit, sound and light isolated Faraday cage. During the EEG recording, the participants were monitored by cameras placed inside the Faraday cage, which provided only momentary monitoring without recording. The participants were informed about this situation in advance.

#### Visuospatial attention paradigm

**Fig. 2:**
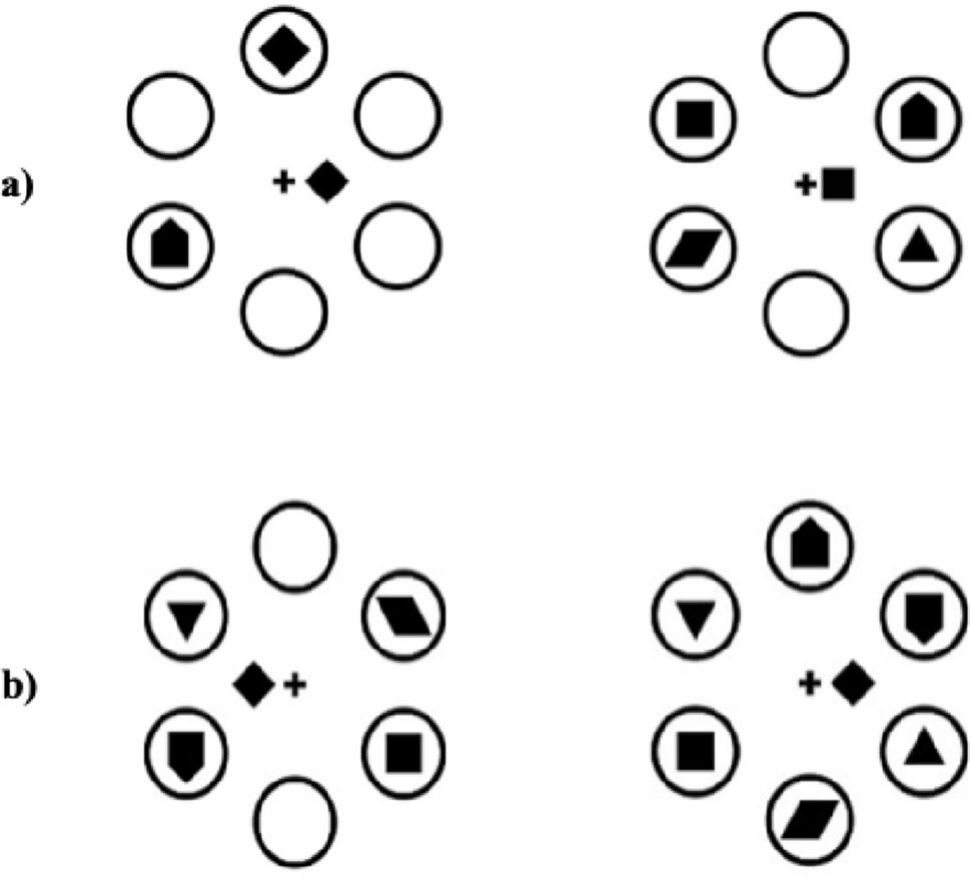
**a** Examples of congruent stimuli in the VSA paradigm. The target stimulus inside the circle and the target stimulus next to the fixation cross (the target stimulus for the left figure is a diamond shape, the target stimulus for the right figure is a square shape) are the same. **b** Examples of incongruent stimuli in the VSA paradigm. The target stimulus inside the circle and the target stimulus next to the fixation cross are different (For the figure on the left, the target stimulus inside the circle is a square, and the target stimulus next to the fixation cross is a diamond. For the figure on the right, the target stimulus inside the circle is a square, and the target stimulus next to the fixation cross is a diamond.)

**Fig. 3:**
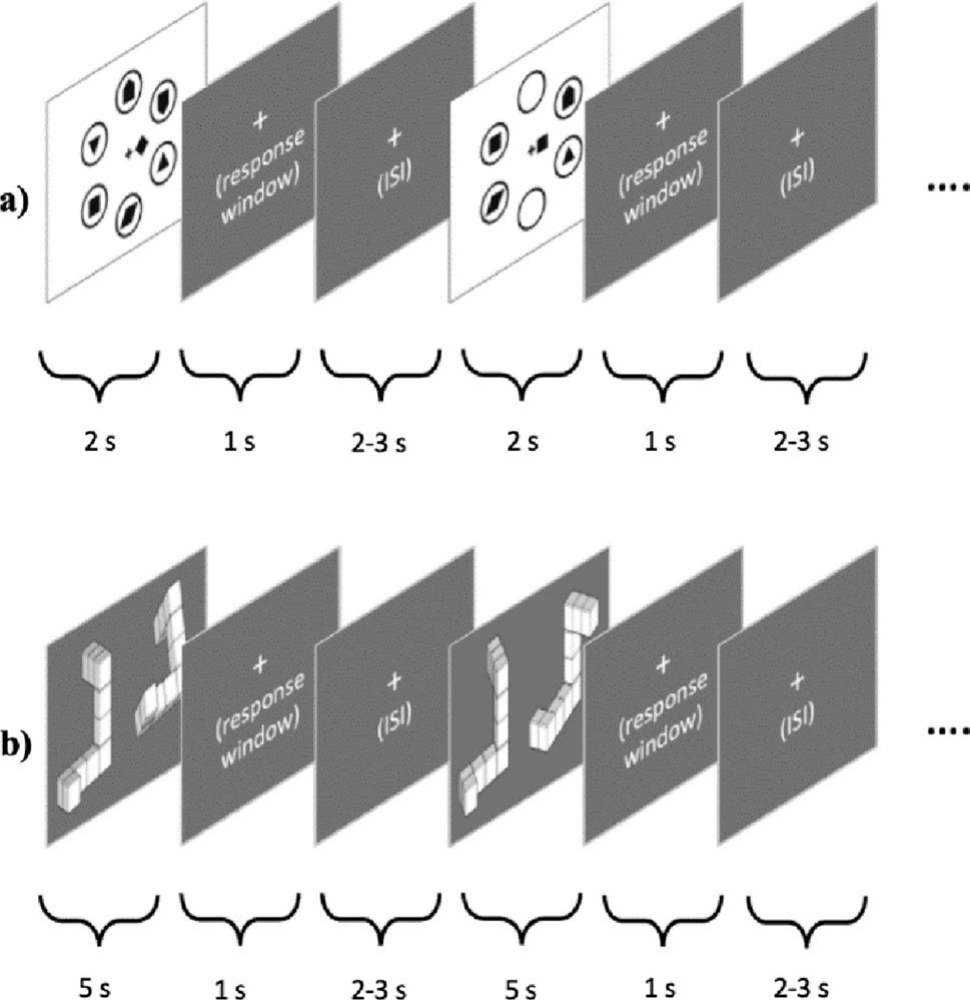
**a** Flow chart of VSA paradigm. **b** Flow chart of MR paradigm

In our study, the experimental design developed by Green and Bavelier (2006), which is used in this field for the assessment of VSA, was taken as an example. In this paradigm, a total of 6 circles and a plus sign in the middle of these circles appear on the screen. There are shapes inside the circles and next to the fixation plus in the middle. A total of 7 different shapes (triangle, inverted triangle, parallelogram, square, pentagon, inverted pentagon and diamond) are randomly positioned. These shapes are randomly assigned inside the circles. All or part of the circles are set to be filled. In addition, a distractor target stimulus (square or diamond) is added next to the fixation plus in the middle of each shape. The subjects were told in advance that the target stimuli were ‘square’ and ‘diamond’ shapes. When each image appeared on the screen, only one target stimulus was inside the circles (either square or diamond). The incoming shapes were divided into two as ‘congruent’ and ‘incongruent’ stimuli. In the congruent stimulus, the distractor shape next to the fixation cross in the middle and the target shape inside the circle are the same (both are either square or diamond) (Fig. 2a). In the incongruent stimulus, the distractor shape next to the fixation cross and the target shape inside the circles are different (one is square, the other is diamond) (Fig. 2b) (Suppl. for application details) (Fig. 3).

#### Mental rotation paradigm

**Fig. 4:**
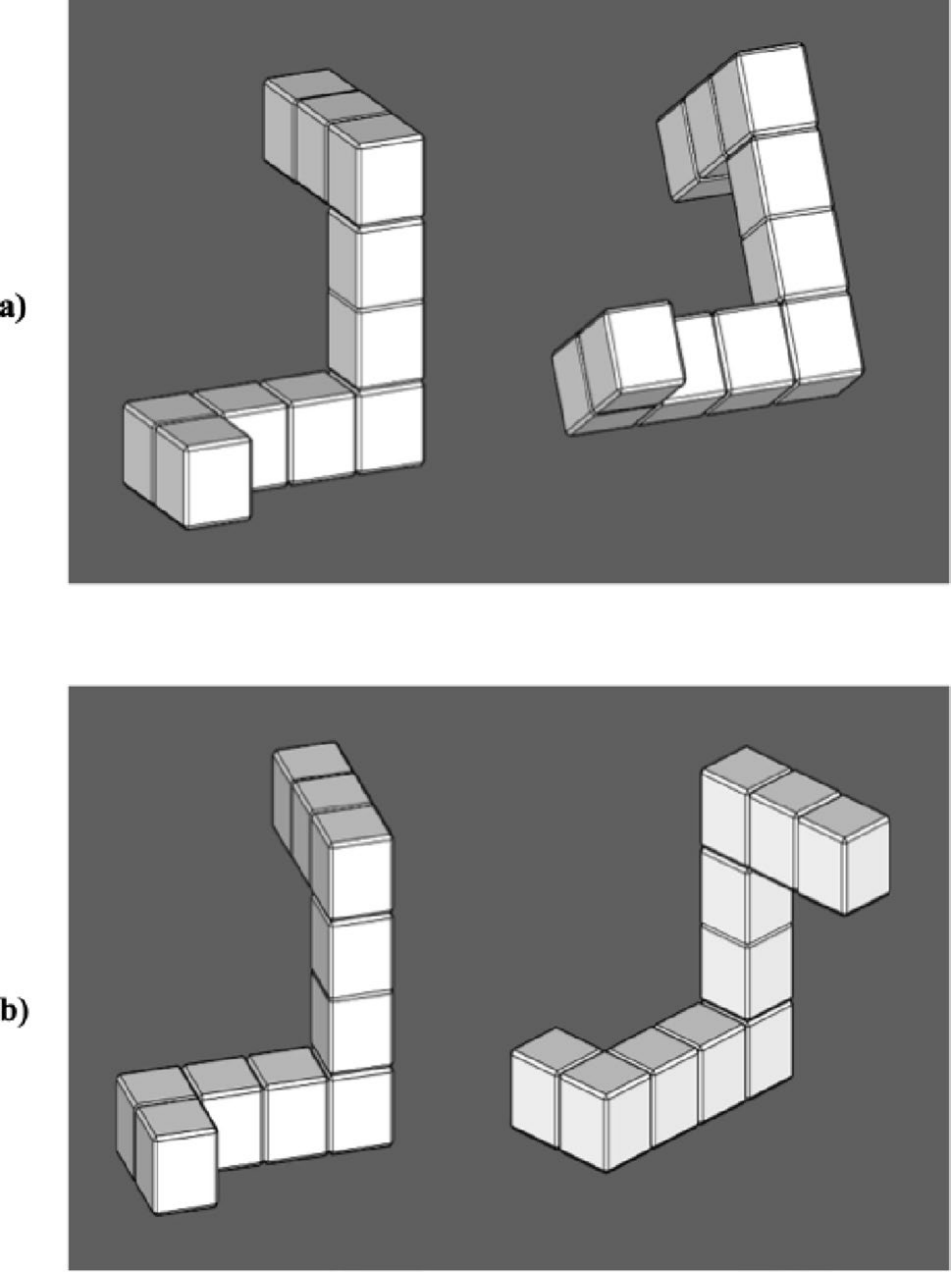
**a** Example of a MR paradigm congruent stimulus. Reference figure (left) and congruent stimulus (right, rotated 30° about the x-axis). **b** An example of an incongruent stimulus in the MR paradigm. The reference figure (left) and the incongruent stimulus (right, the mirror-symmetric version of the reference figure rotated 150° on the y-axis)

A paradigm was created by using the library consisting of 18,000 images in total, as described in the study of Peters and Battista (2008). Shading, color contrasts and depth perceptions were added to the shapes to increase the perception of 3D. The shapes to be used in the test were made in the Sketch-Up program. A three-dimensional shape created from a total of ten identical cubes was used as a reference (left figure). In each stimulus, there would be a rotation of this reference shape in the x, y and z axes and a second shape like this shape rotated in the x, y and z axes on the right side (right figure). This second shape consisted of the rotation of the reference shape in only one of the x, y and z axes (congruent stimulus) (Fig. 4a) or the rotation of the symmetry of the reference shape (incongruent stimulus) (Fig. 4b). The rotation angles of the shapes relative to each other were determined as: 30°, 60°, 90°, 120°, 150° (Suppl. for application details) (Fig. 3).

The paradigms were applied to the participants consecutively during pre- and post-EEG recordings. Randomization was applied within the subjects for both paradigms between pre- and post-exams. Participants who were first applied the VSA paradigm in the pre-recording, solved the MR paradigm first in the post-recording. Participants who were first applied the MR paradigm in the pre-recording, solved the VSA paradigm first in the post-recording. Considering that the participants would solve the paradigms consecutively, fatigue could affect performance, and such a balancing was performed.

### EEG analysis

#### Analysis of event-related oscillations in time and frequency domains

Brain Vision Analyzer (Brainproduct, Munich, Germany) program was used for the analysis of event-related EEG data. Raw EEG data recorded during the VSA task were analyzed separately for correct responses to congruent and incongruent stimuli. Responses to congruent stimuli were analyzed during the MR task. In the analysis of raw EEG data, pre-processing stages consisting of digital filter (IIR filters; 0.01–60 Hz), independent component analysis (ICA) for extracting components of eye blinks, right-left eye movements and muscle artefacts, segmentation and denoising were performed. In segmentation, the VSA paradigm was segmented into epochs as 2000 ms before the stimulus and 3000 ms after the stimulus (−2000 3000 ms) for correct responses. Segmentation for the MR paradigm was as follows; for correct responses, it was divided into epochs as 2000 ms before the stimulus and 7000 ms after the stimulus (−2000-7000 ms). Then, artifacts such as eye movements, blinks and muscle movements that appeared in the EEG were cleaned. Advanced analyses, namely Event-Related Power Spectrum analyses, were performed on the noise-cleaned recordings for each trial series.

#### Event-related power spectrum (ERPS) analysis

One of the time-frequency analyses is the event-related power spectrum analysis. The ERPS analysis evaluates how much the response to the stimulus changes in spectral density compared to the pre-stimulus when a stimulus is given. In our study, the event-related power spectrum analysis were be performed using the wavelet transform for theta and alpha frequency bands. For this, wavelet transform (Continuous Wavelet transform, Gabor normalization) was applied to each epoch in the pre-processed EEG data. Wavelet analysis: It was done for theta (4–7 Hz; 3 cycle) and HA (11–13 Hz; 3 cycle) frequency bands. When applying Wavelet in the event-related power spectrum analysis, a certain time interval before the stimulus (−500 and − 300 ms interval for theta and alpha) was subtracted from the post-stimulus responses (Baseline correction). Then, the average of all epochs to which Wavelet was applied was taken. Thus, the event-related power spectrum in decibels (dB) was obtained in the time-frequency plane. Each frequency band of the data to which Wavelet was applied and averaged was calculated by looking at the grand averages for correct responses, and these values were used in the statistical analysis.

#### Behavioral data analysis

The number of correct responses to VSA and MR paradigms was recorded as behavioral data. In the literature, the time it takes for individuals to respond to stimuli is also referred to as reaction time. In these studies, individuals can press the response at any time after viewing and deciding on the stimuli. After the stimulus arrives, individuals need motor planning to navigate to the correct response and motor movement to press the correct response. Although these cognitive and motor processes may appear brief, they lead to distinct processes in EEG data. Cortical activity during movement has received considerable study to date. It is thought that during voluntary hand movements, the beta (15–28 Hz) and mu (8–13 Hz) rhythms change and act as part of the process (Pfurtscheller and Lopes Da Silva 1999). The desynchronization of mu and beta rhythms begins contralaterally 1.5 s before the movement, and after the movement is executed, synchronization is replaced by desynchronization; this is considered to reflect cortical deactivation. This occurs slightly earlier in the beta band than in the mu band(de Jong et al. 2006). This information can lead to unintended data in the EEG data of motor planning and motor movement. In our study, we chose to place a post-stimulus response screen to prevent the EEG data from being affected by motor planning and motor movement processes. Therefore, subjects were unable to press the response when they wanted. Therefore, we did not include reaction time as a behavioral data.

### Statistical analysis

Statistical analysis was performed using SPSS 22.0 (Statistical Package for Social Sciences) and Jamovi 2.5.27. programs. In this study, the normality of the distribution was examined using the Shapiro-Wilk test. Repeated measures ANOVA was used for each paradigm separately, for each event-related oscillation analysis. Independent samples T test was used to compare the initial demographic data between groups. Mann-Whitney U test was used to examine whether there was any difference between the number of correct responses given to paradigms. During ANOVA analysis, for comparison between groups, 2 groups (VBBEG, SBEG) were entered as factor levels. In intra-group comparisons, time; 2 factor levels before and after the application, hemisphere; 2 factor levels as right and left, and analyses were performed for location with at least 3 factor levels. For results with significant time*group interaction, paired samples T test was used to determine which group the significance stemmed from before-after difference. Wilcoxon test was used for statistical comparison of intra-group pre- and post-paradigm correct responses. The significance level was accepted as *p* < 0.05 for all comparisons. Greenhouse-Geisser correction was applied during the ANOVA test. Bonferroni correction was applied in post-hoc tests.

## Results

Of the 34 participants included in the study, 3 participants were excluded from the study 1, participant also left the study. A total of 30 participants completed the study.

### Demographic data

A demographic information form was filled out when the individuals were included in the study. Within the scope of the information received, the gender, age, height, weight and education period of the participants are shown in Table 1. As a result of the comparison of demographic data between the groups, there was no statistically significant difference between age, gender, height, weight and years of education.

**Table 1:** Demographic Information of Participants

	SBEG	VBBEG	*p*
	Mean ± SD (*n*/%)	Mean ± SD (*n*/%)	
Age	24.7 ± 4.42	25.6 ± 3.85	0.994
Gender (women/men)	8 (%53.4)/7 (%46.6)	8 (%53.4)/7 (%46.6)	
Height	171 ± 8.84	171 ± 9.49	0.969
Weight	67.8 ± 11.3	67.9 ± 16.4	0.980
Education period	16.7 ± 3.09	17.3 ± 2.69	0.533

*SBEG* Structured Balance Exercises Group, *VBBEG* Video-based Balance Exercises Group, *SD* Standard Deviation, *n* number, %: rate

### Results of behavioral data

#### Initial comparison of behavioral data

In the VSA paradigm, no statistically significant differences were observed between the SBEG and VBBEG groups in the number of correct responses for either congruent (*p* = 0.343) or incongruent stimuli (*p* = 0.678). Likewise, in the MR paradigm, correct response rates did not differ significantly between groups (*p* = 0.967).

#### Comparison of behavioral data before and after treatment within groups

In the SBEG group, no significant differences were found between pre- and post-test scores for correct responses to congruent and incongruent stimuli in the VSA paradigm (*p* = 0.068). In contrast, the number of correct responses in the MR paradigm was significantly higher in the post-test compared to the pre-test (*p* < 0.001). These findings indicate that MR performance significantly improved following the 6-week exercise program.

When the number of correct responses given to the congruent stimulus in the VBBEG VSA paradigm was compared, there was no statistically significant difference between pre-measurement and post-measurement (*p* = 0.057). When the number of correct responses given to the incongruent stimuli was compared as pre and post, again no significant difference was found (*p* = 0.093). When the correct responses given to the MR paradigm were compared, there was a significant difference between pre and post measurements, as in the SBEG (*p* = 0.038).

### Results of EEG Data

#### Results of theta power analysis of VSA paradigm

**Fig. 5:**
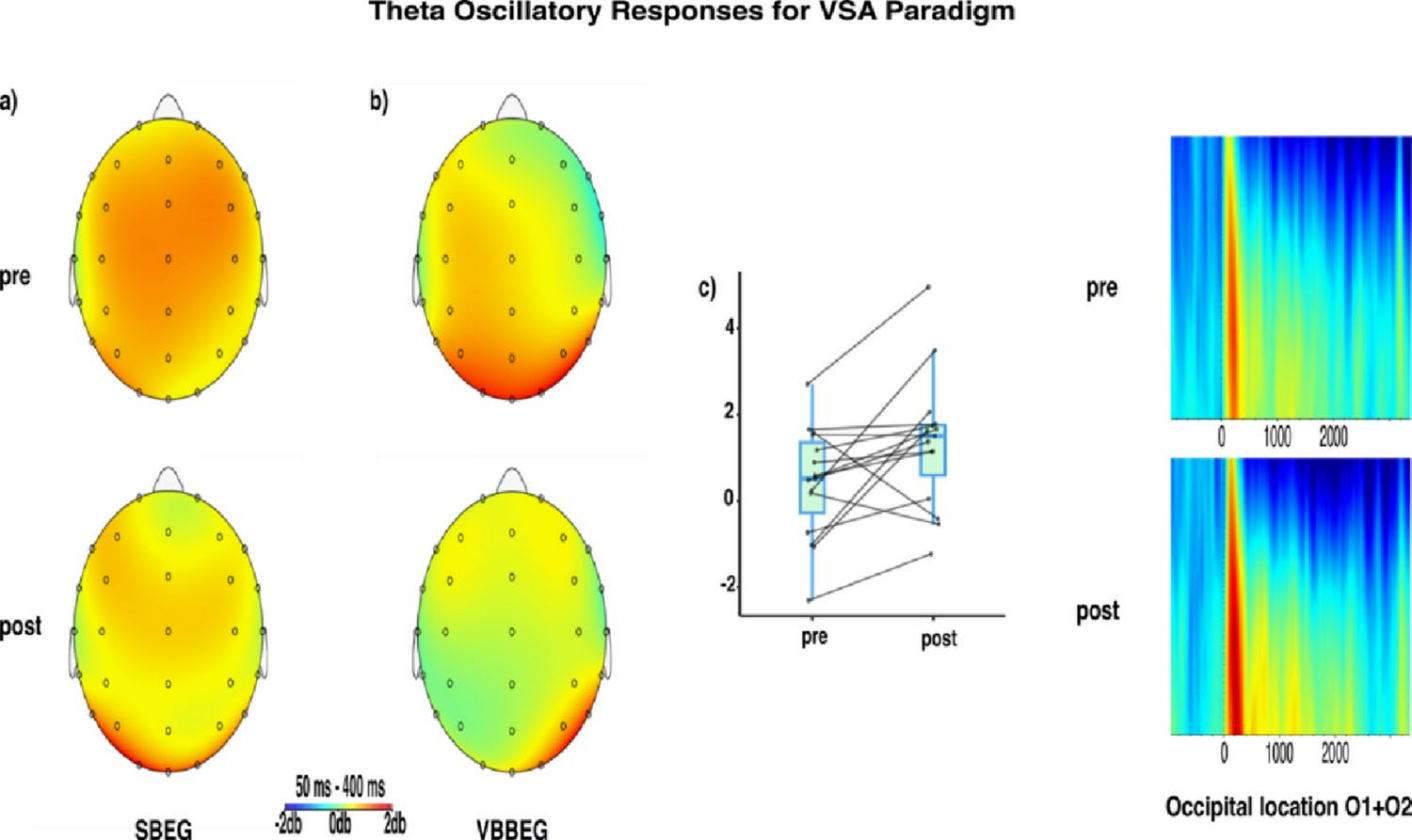
Pre and post topographic map view of theta (4–7 Hz) oscillations compatible stimulus in the VSA paradigm. **a** Pre (top) and post (bottom) topographic map view for SBEG. **b** Pre (top) and post (bottom) topographic map view for VBBEG. **c** VSA paradigm congruent stimulus theta power analysis SBEG occipital location pre-post comparison. SBEG pre (Mean = 0.836, SD = 1.05), post (Mean = 1.354, SD = 1.57) statistical comparison (*p* = 0.244) (left). SBEG occipital location pre (top)-post (bottom) theta oscillation (4–7 Hz) grand average Wavelet plots (right)

To determine the effects of six weeks of training in the VSA paradigm, pre- and post-event-related theta oscillation (4–7 Hz) responses were examined for congruent and incongruent stimuli. The topographic view of theta oscillations in the VSA paradigm is shown in Fig. 5a-b.

*Results of congruent stimulus EEG analysis*: 2 (group: SBEG, VBBEG) X 2 (time: pre, post) X 2 (hemisphere: left, right) X 4 (location: frontal, temporal, parietal, occipital) designed as repeated measures ANOVA test was examined. As a result of this analysis; group*time*location (F(2.29, 64.02) = 3.87, MSe = 5.48, *p* = 0.021, η2p = 0.122) interaction was found significant. In order to find out which location the significance originated from, 2 (group: SBEG, VBBEG) X 2 (time: pre, post) repeated measures ANOVA test was performed for each location. As a result of these measurements, significant results emerged only for occipital location theta oscillatory responses (F(1, 28) = 4.60, MSe = 5.33, *p* = 0.041, η2p = 0.141).

Paired Samples T-test was performed to find out which group’s pre-post comparison caused this significance in occipital location. No statistical significance was found in the SBEG group pre (Mean = 0.836, SD = 1.05), post (Mean = 1.354, SD = 1.57) comparison (*p* = 0.244, dz = 0,31) (Fig. 1c). When VBBEG pre (Mean = 1.602, SD = 2.53, dz = 0.31) and post (Mean = 0.940, SD = 1.92) results were compared, no significant difference was found for occipital location (*p* = 0.078, dz = 0,49).

*Results of incongruent stimulus EEG analysis results*: Repeated measures ANOVA test designed as 2 (group: SBEG, VBBEG) X 2 (time: pre, post) X 2 (hemisphere: left, right) X 4 (location: frontal, temporal, parietal, occipital) was analysed.

As a result of the analysis, group*time*location interaction (F(3, 84) = 4.56, MSe = 5.52, *p* = 0.005, η2p = 0.140) was found to be significant. In order to determine which location, the time*group interaction was specific to, a separate 2 (group: SBEG, VBBEG) X 2 (time: pre, post) repeated measures ANOVA test was performed for each location. As a result of this second location-specific repeated measures ANOVA test, it was determined that the significance was specific only to the occipital location (F(1,28) = 20.5, MSe = 12.70, *p* = 0.003, η2p = 0.273).

To find out which group this significance in occipital location is due to pre-post comparison, pre-post measurements of each group were compared with Paired Samples T test. When SBEG pre (Mean = 0.426, SD = 1.29) and post (Mean = 1.354, SD = 1.55) results were compared, there was a significant difference for occipital location (*p* = 0.025, dz = 0.62).

VBBEG group occipital location theta power pre (Mean = 1.709, SD = 2.21) and post (Mean = 0.795, SD = 2.28) comparison did not find a significant result (*p* = 0.052, dz = 0.52).

#### Results of alpha power analysis of MR paradigm

**Fig. 6:**
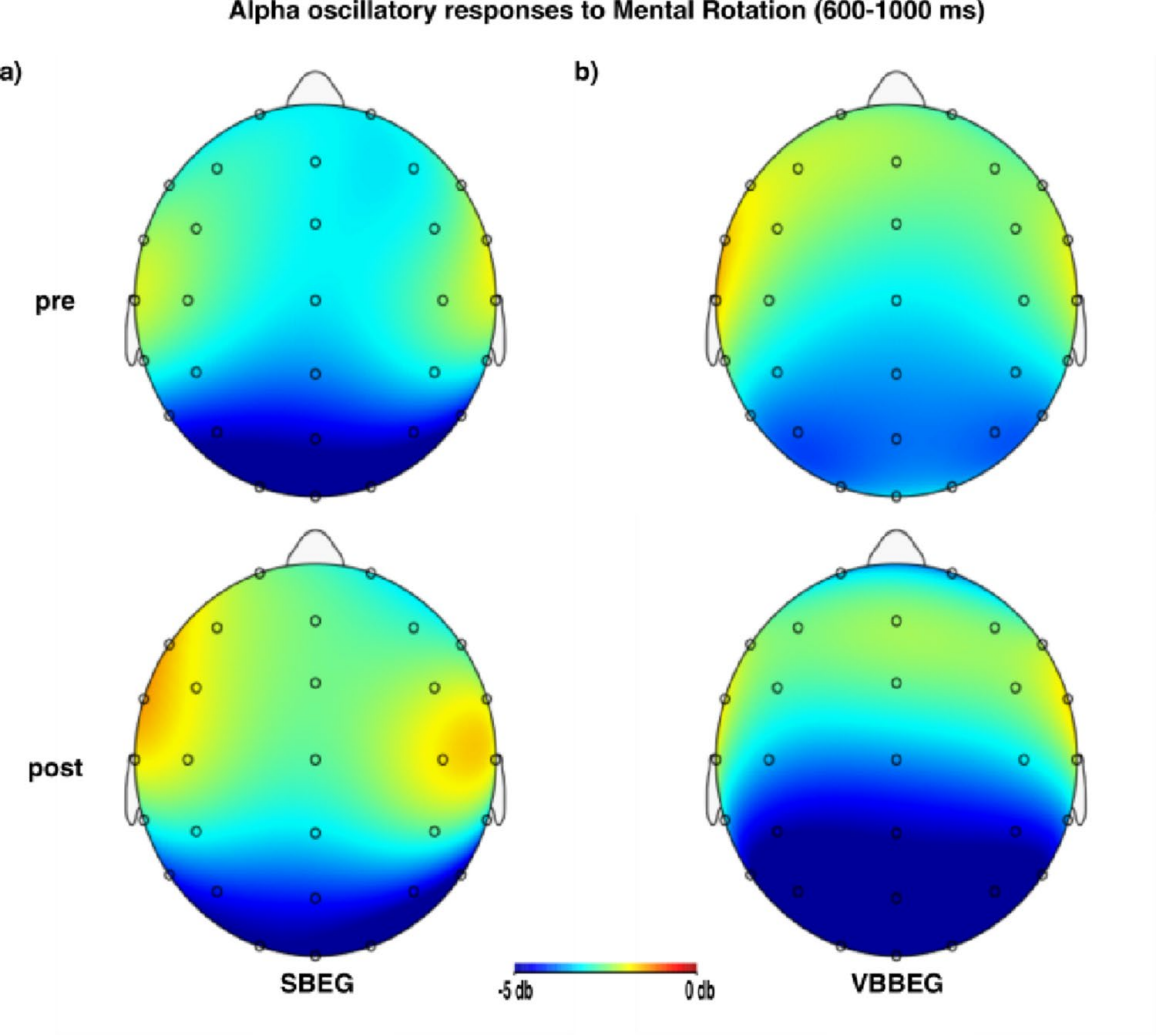
Pre and post topographic map view of 600–1000 ms HA (11–13 Hz) oscillations in the MR paradigm. **a** Pre (top) and post (bottom) topographic map view for SBEG. **b** Pre (top) and post (bottom) topographic map view for VBBEG

**Fig. 7:**
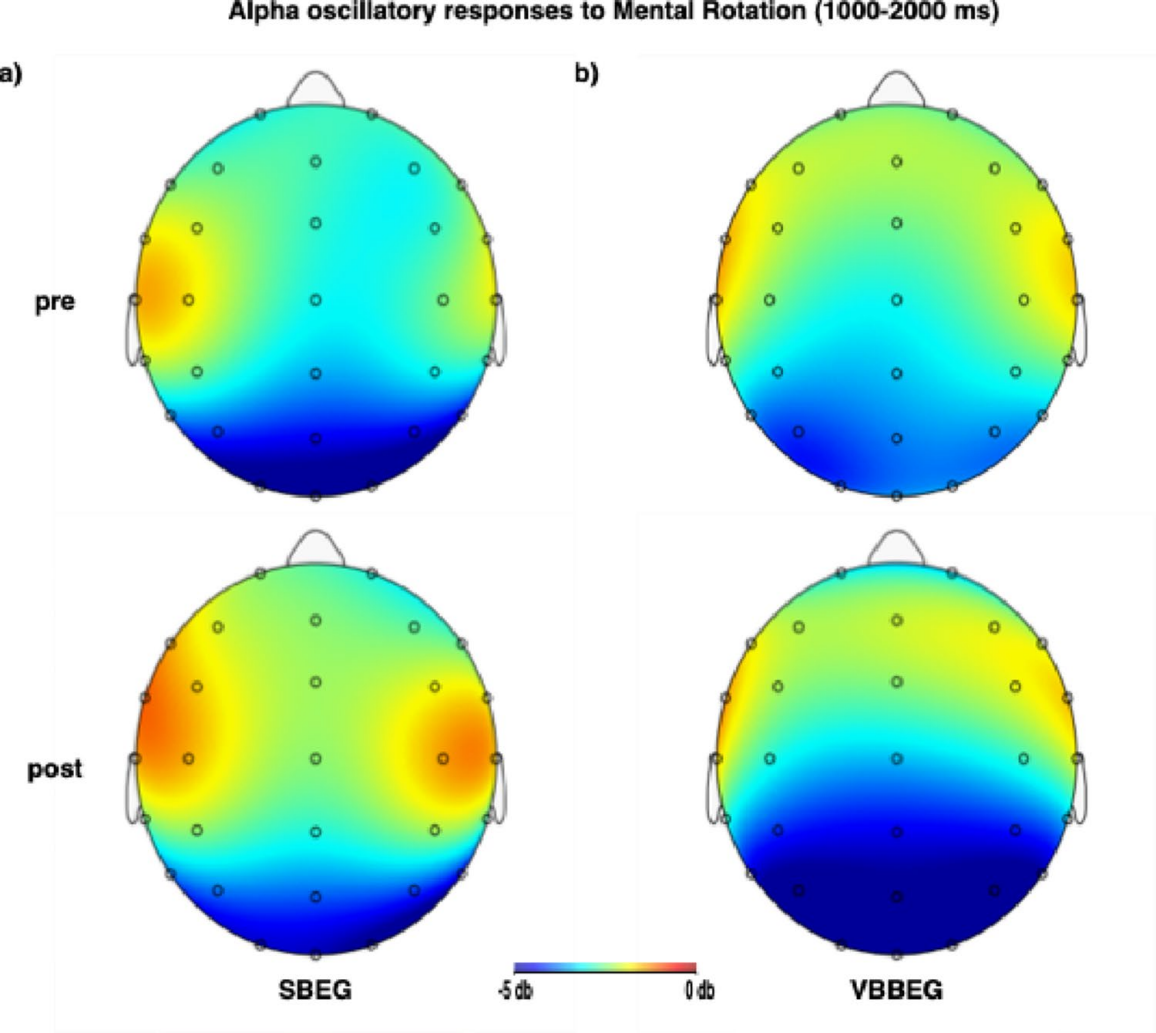
Pre and post topographic map view of 1000–2000 ms HA (11–13 Hz) oscillations in the MR paradigm. **a** Pre (top) and post (bottom) topographic map view for SBEG. **b** Pre (top) and post (bottom) topographic map view for VBBEG

To determine the effects of 6 weeks of exercise in the MR paradigm, pre and post event-related HA oscillation responses were examined for congruent stimuli. To determine the effects of 6-week exercise programs on the MR task; a repeated-measures ANOVA test designed as 2 (group: SBEG, VBBEG) X 2 (time: pre, post) X 2 (hemisphere: left, right) X 3 (location: central, centroparietal, parietal) was applied. This analysis was applied for 2 different time windows. The selected time windows were 600–1000 ms (Fig. 6) and 1000–2000 ms, which are consistent with the literature (Fig. 7). The time windows for the mental rotation paradigm were selected in accordance with the literature (Ozga et al. 2018).

In the 600–1000 ms time window alpha EEG analyses, the time*group interaction (F(1, 28) = 4.56, MSe = 53.48, *p* = 0.041, η2p = 0.140) was found to be significant. To investigate which, group this interaction was due to, the pre-post comparison, and the pre-post and post measurements of each group were examined within themselves by applying a Paired Samples T test.

SBEG 600–1000 ms alpha power analysis results: In the 600–1000 ms alpha power EEG analysis result, no statistical significance was found in the pre (Mean = −3.43, SD = 2.19) and post (Mean = −3.01, SD = 2.61) comparison (*p* = 0.416, dz = 0,21).

VBBEG 600–1000 ms alpha power analysis results: 600–1000 ms alpha power EEG analysis results Pre (Mean = −3.38, SD = 2.64) and post (Mean = −4.24, SD = 2.06) measurements were compared for VBBEG. The result was statistically significant (*p* = 0.035, dz = 0,58).

When the time*group interaction (F(1, 28) = 2.72, MSe = 27.23, *p* = 0.110, η2p = 0.089) was examined in the 1000–2000 ms time window alpha EEG analyses, no significant difference was found (Fig. 6.18). However, when the analysis table was examined, the groups showed parallel changes with the 600–1000 ms analyses.

## Discussion

This study investigated the effects of two different balance exercise interventions—video-based balance games and structured balance exercises—on VSA and MR abilities in healthy participants through EEG brain oscillation analysis. The cognitive functions assessed in this study were measured using tasks previously used in the literature. Following the six-week intervention period, a reduction in occipital theta power was observed in the VBBEG during the VSA task, whereas an increase was noted in the SBEG. Regarding the MR task, HA power in centroparietal regions decreased in the VBBEG and increased in the SBEG. It has been observed that two different types of exercise can affect electrophysiological processes in cognitive tasks in different ways. However, the distinct modulation of brain oscillatory activity suggests that each type of exercise exerts its effects through different neurophysiological mechanisms.

Although the VBBEG demonstrated higher post-test scores for congruent and incongruent stimuli in the VSA paradigm, the differences compared to pre-test values were not statistically significant. When the VBBEG was examined, no statistically significant difference was found between congruent and incongruent stimuli pre-post in the VSA paradigm. Green and Bavelier (2006) also studied reaction times in their study but did not provide information on the number of correct responses. Another study compared selective attention and working memory through reaction times (Lavie et al. 2004). Studies have highlighted the differences in neural control mechanisms between congruent and incongruent stimuli, which influence reaction time. We did not expect any difference in the number of correct responses using this paradigm. Our goal was to investigate changes in spatial search for congruent and incongruent stimuli and to demonstrate how training affected these processes. The number of correct responses analyzed for the MR paradigm was compared for each group as pre- and post-exercise. The results showed an increase in the number of correct responses in the MR paradigm in both groups after the intervention. The statistical improvement was observed in favor of the SBEG. Jansen et al. (2010), in their study comparing those who did physical activity and those who did not, found that those who did physical activity had more MR correct responses. Jost et al. (2023) wanted to examine the effect of exercise on MR simultaneously. They applied a MR test to the participants during aerobic exercise. They found that as the duration of cycling increased, the number of correct responses in the MR task increased. In their study examining the effects of balance exercises on elderly individuals, Rogge et al. (Rogge et al. 2017) found significant increases in longitudinal cognitive skill scores. Our research has not found any study investigating the effectiveness of balance-based exercises on MR ability. However, considering the information we shared from the literature regarding the relationship between physical activity and MR, the exercises we implemented increased the number of correct responses on the MR task. Our study is parallel to the literature in this respect.

Before moving on to the discussion part regarding the VSA paradigm, to the best of our knowledge, no previous study has been found that has applied the paradigm we used in our study using EEG. The EEG data of the VSA paradigm were analyzed separately for congruent and incongruent stimuli. Theta oscillations were the most frequently studied frequency range in the literature regarding the VSA task. However, as a reminder, converging evidence regarding spatial attention indicates that environmental sampling, whether through covert or overt mechanisms, is fundamentally a rhythmic process and has a sampling rate in the theta band (Fiebelkorn et al. 2018). When we examined the results of the congruent stimulus analysis, the group*time*location interaction was found to be significant. The main effect of location was found to be significant only for the occipital location. As a result of further analyses, the comparisons were not significant for both groups but were very close to significance for the VBBEG group. Another notable situation is that after 6 weeks of exercise, event-related occipital theta oscillatory responses increased in the SBEG, while they decreased in the VBBEG. A significant group*time*location interaction was found again when compared with the incongruent stimulus. The location main effect was significant for the occipital region. When both groups were compared pre- and post-compared, event-related theta oscillatory responses increased significantly in the SBEG. In the VBBEG, this was not statistically significant but decreased very close to significance after the exercise program. In summary, for SBEG, oscillatory theta (4–7 Hz) responses to congruent and incongruent stimuli increased, while they decreased in the VBBEG. In light of this information, we can say that the two different exercise applications affect event-dependent oscillatory theta responses differently during the VSA task. Although hemisphere was a factor in our analyses, no hemispheric differences were found. The frontal and parietal areas within the Dorsal Attention Network system are responsible for determining the areas to which attention is directed (Hopfinger et al. 2000). In the paradigm we used, target stimuli originated from the screen’s left, right, or center. This is because the epochs included in the analyses included all stimuli originating from the left, right, and center. In various detection and discrimination paradigms, voluntary or endogenous visual selection has been found to activate the dorsal parietal and frontal cortices most consistently (Corbetta 1998). Our study also observed activation in these areas, but no statistically significant difference was found. Local enhancement of oscillatory activity in the early visual cortices has been linked to visual processing and perceptual grouping by intracranial recordings. The occipital cortex has been implicated in visual processing in general and perceptual grouping in particular (Castellano et al. 2014). Along with activation in the frontal and parietal areas, transient activation in the occipital cortex has been demonstrated in fMRI studies. It is thought that the transient response in the occipital region may reflect the processing of the visual stimulus (Corbetta and Shulman 2002). Although areas such as the temporal and parietal regions are involved in the early stages of visual processing, the extracitriate cortex, particularly area V4, is frequently activated when attention is directed to or memory is desired for a stimulus in the receptive field(McAdams and Maunsell 1999). Furthermore, the extracitriate cortex, also located in the occipital lobe, is known to be activated during object discrimination (Corbetta 1998). Dugué et al. (2015), in their study examining the neuronal mechanisms of a visual search task, found that theta phase activity after stimulation increased in favor of correct responses, with this increase peaking at 6.6 Hz. They stated that this increase was valid for the occipital, parietal and frontal regions. In a study investigating the role of visual areas in redirecting voluntary attention using transcranial magnetic stimulation (TMS), it was concluded that theta frequency was deactivated in the early visual area in the occipital lobe using TMS and that spatial attention was impaired. Based on this information, the study suggested that the occipital theta frequency functions in directing spatial attention (Dugué et al. 2016). Green and Bavelier (2006) compared the VSA abilities of action video game players and non-players. The assessment was conducted using the same paradigm we used. They concluded that video games positively affected VSA skills, as the reaction times to the paradigm stimuli were shorter in action video game players. Video games have been reported to have unusually high attentional demands, much greater than those a person might be exposed to daily. It has also been reported that multiple elements must be processed simultaneously during video games. Considering this information, we believe that the group receiving video-based balance exercises facilitated visual processing in the occipital region through theta oscillations compared to structured balance exercises, considering the attentional elements they were exposed to due to the video-based games, in addition to the exercise itself. We attribute the theta decrease in the video-based balance exercises group to the decreased cognitive load in response to the facilitation of visual processing. We attribute the lack of statistical significance of the decrease in VBBEG to the small number of participants. We believe that significance will be achieved with an increase in the number of participants. When we examine the changes in occipital theta oscillatory responses in the structured balance exercises group, a statistically significant increase in occipital theta oscillatory power is striking. Only a few researchers in the literature have studied posterior theta activity. One of these studies suggested that early theta activity may also be involved in the inhibition of non-target stimuli. For stimuli randomly presented on only one half of the screen, an ipsilateral theta increase was found to the opposite stimulus, followed immediately by a contralateral theta increase (Lubbe and Utzerath 2013). Another study examining early posterior theta activity involved the selection of target and non-target-colored shapes and found an occipital theta increase ipsilateral and contralateral to the target stimulus after the stimulus (Van Der Lubbe et al. 2014). Haciahmet et al. (2023), in their study using congruent and incongruent distractors, found that the contralateral theta increase was greater for incongruent distractors compared to congruent distractors. Our study differed from these shared studies in terms of the direction of the stimulus. In our paradigm, stimuli were arriving from all directions. Therefore, we believe that, unlike these studies, bilateral theta increases occurred. Considering all this information about theta oscillations, theta activity during selective attention is thought to be related not only to the selection of the desired stimulus but also to the direct suppression of irrelevant stimuli (Asanowicz et al. 2023). Considering this information, we believe that the inhibition ability in the occipital area increased in the group receiving structured balance exercises. Finally, both exercises appear to have the same effect on the processes of two different stimuli in a VSA task. Considering the difference in stimulus, no difference in the effects of the exercises was observed. Therefore, congruent and incongruent stimuli were not discussed separately.

According to the EEG analysis results of the MR paradigm, the comparison of pre- and post-VBBEG results in the 600 − 100 ms HA oscillatory responses was found to be statistically significant. There was no significance in the 1000–2000 ms results, but when changes were observed, alpha event-related desynchronization (ERD) increased in the VBBEG and decreased in the SBEG for both time windows. In our study, a MR task involving manipulation of a reference shape consisting of ten equal cubes was applied, based on the library created by Peter and Battista (2008). In the literature, the MR task has been associated primarily with alpha/beta ERD based on oscillations. Riečanský and Katina (2010), in their study assessing MR using EEG brain oscillations, interpreted MR ability based on the alpha oscillations (8–13 Hz) and reaction times they reported in their analysis. They noted that those with faster reaction times had less alpha ERD over the parietal cortex. They attributed this to decreased cognitive load. According to the neural efficiency hypothesis, increased cognitive demand increases alpha ERD. Accordingly, alpha ERD decreases when the task is easier to perform (Riečanský and Jagla 2008). Another perspective related to MR is its relationship with motor planning (Tomasino and Gremese 2016). Motor planning can be explained as the processes that enable collecting and integrating information about actions to embody a targeted movement (Hoshi and Tanji 2007). It has been reported that the increase in motor planning ability increases in direct proportion to MR skill (Tomasino and Gremese 2016). The relationship between motor processes and MR is not limited to this. During a MR task, subjects have been reported to imagine and execute the rotation process as if they were rotating with their hands. This demonstrates that MR processes act in conjunction with not only mental but also physical processes (Gardony et al. 2017). EEG oscillations (8–13 Hz) in the sensorimotor cortex associated with motor movements are called mu rhythms. It has been reported that the decrease in mu rhythm power in the sensorimotor cortex occurs in situations such as motor stimulation as well as in the preparation and execution of movements (Pfurtscheller and Lopes Da Silva 1999; Llanos et al. 2013). Furthermore, the decrease in mu power is thought to reflect decreased intracortical inhibition and increased pyramidal neuron firing in the primary motor cortex (Gardony et al. 2017). Chen et al. (2013) found a decrease in mu power in the sensorimotor and parietal cortices during a MR task using hand photographs. This decrease was attributed to the participants attempting to match the hand images on the screen with their own hands by rotating them mentally. In our results, we also demonstrated this decrease with electrodes located on the sensorimotor cortex. This decrease in the sensorimotor cortex was present in both groups at the beginning of the study. After exercise, these decreases increased in the participants in the VBBEG, while they decreased (i.e., shifted positively) in the SBEGG. Another important area related to MR is the parietal cortex. The parietal cortex has been shown to be involved in mental visuospatial representation and is active in action-oriented mental image formation and maintenance tasks (Zacks 2008; Sasaoka et al. 2014). These findings are also supported by fMRI studies showing increased hemodynamic responses in the parietal lobe during a MR task (De Lange et al. 2005; Gogos et al. 2010). A HA focus has been reported to provide higher spatial focus (Horst et al. 2013). Changes in HA power have also been shown to provide a valid means of revealing the relationship between the involvement of visual and motor processes and the task (van der Helden et al. 2010). In our study, we chose the 11–13 Hz range for alpha analysis. Horst et al. (2013), evaluated MR using hand images and found decreases in HA power in the central, centroparietal, and occipital regions during the task. Reiner (2016) found significant improvements in MR task performance in participants who received HA (10–12 Hz) neurofeedback training compared to participants who did not receive neurofeedback training. This result led them to argue that HA frequency is directly related to MR. Surprisingly, we found a finding that contradicts the information shared so far in the literature. In the group that received video-based balance exercises, a significant increase in HA ERD was observed after a 6-week exercise program, accompanied by improved task performance. This is noteworthy as it contradicts and is not previously reported in the literature. The literature currently suggests that ERD is directly proportional to increasing cognitive load. This important finding should be investigated in future studies. When comparing video-based balance exercises and structured balance exercises, we believe that the exercises in the SBEG fostered motor planning processes more strongly. This is because the exercises performed in this group involved numerous challenges, such as increasing and decreasing the support base and changing from a stationary support base to a moving support base. Furthermore, performing the exercises with eyes open and closed may have influenced motor planning. In contrast, the video-based balance exercises did not involve processes such as changing the support base or moving the ground. In our study, after a 6-week exercise program, alpha ERD increased in the VBBEG group, while alpha ERD decreased in the SBEG. We can associate the HA increase in the VBBEG with visualization during the task. Subjects were exposed to many visual stimuli during the video-based balance games. As a result, we interpret that mental representations of shapes were better formed during the MR task. Interpreting our results in terms of alpha ERD, we can say that structured balance exercises modulate MR ability better than video-based balance exercises.

To our knowledge, this is the first study to examine the effects of a six-week balance exercise program on VSA and MR, but it has some limitations that should be considered. The visual paradigms employed included similar pre- and post-test stimuli, and participants were exposed to a total of 200 images during the initial assessment. Although they are unlikely to have retained these images over the six weeks, a potential learning effect cannot be entirely ruled out. Moreover, the study focused on comparing two different exercise interventions in terms of their effects on these cognitive functions; however, the absence of a non-exercising control group limits the strength of the conclusions that can be drawn. Furthermore, in our study, the stimulus window and the response window were independent of each other. This prevented us from obtaining reaction time data. The lack of reaction time data creates limitations in terms of interpreting behavioral data. The exact ovulatory phase was not objectively verified by hormonal measurements, which may be considered a limitation. However, based on menstrual cycle tracking, participants were expected to be predominantly in the early luteal phase, characterized by increased progesterone levels. We believe that future studies should consider this aspect to gain a more comprehensive understanding of the mechanisms involved.

## Conclusion

Theta oscillations were observed during the VSA task; post-intervention theta power increased in SBEG and decreased in VBBEG. This suggests that the two exercises affect VSA through different mechanisms. In the MR task, HA (11–13 Hz) power increased in VBBEG and decreased in SBEG; this may indicate improved motor control in SBEG and increased mental imagery in VBBEG. In conclusion, both exercises appear to affect MR ability in different ways. Longer interventions, reaction time measurements, and brain connectivity analyses are recommended to confirm the findings.

## Supplementary Information

Below is the link to the electronic supplementary material.

Supplementary Material 1

## Data Availability

The datasets used and analysed during the current study are available from the corresponding author on reasonable request.
