# Supplementary material for: Investigation of the effects of balance exercises on visuospatial skills using EEG brain oscillations
Source: Cogn Neurodyn. 2026 Jun 26;20(1):116. doi: 10.1007/s11571-026-10494-4 (PMC13309604; doi:10.1007/s11571-026-10494-4)
Supplement: Supplementary file 1 — Supplementary Material 1 [file 11571_2026_10494_MOESM1_ESM.docx]

**INVESTIGATION OF THE EFFECTS OF VIDEO-BASED BALANCE GAMES AND BALANCE EXERCISES ON VISUO-SPATIAL SKILLS USING EEG BRAIN OSCILLATIONS**

Yasin Yıldırım¹^,5^*, İrem Yemeniciler², Devrim Tarakcı³, Bahar Güntekin⁴^,6^

¹ Istanbul Gedik University, Faculty of Health Sciences, Department of Physiotherapy and Rehabilitation, Istanbul, Turkey

e-mail: [yasin.yildirim@gedik.edu.tr](mailto:yasin.yildirim@gedik.edu.tr)

² Istanbul Medipol University, Institute of Health Sciences, Department of Neuroscience, Istanbul, Turkey

e-mail: iremyemeniciler97@gmail.com

³ Istanbul Medipol University, Faculty of Health Sciences, Department of Ergotherapy, Istanbul, Turkey

e-mail: dtarakci@medipol.edu.tr

⁴ Istanbul Medipol University, Research Institute for Health Sciences and Technologies (SABITA), Neuroscience Research Center, Clinical Electrophysiology, Neuroimaging and Neuromodulation Lab, Istanbul, Turkey

^5^ Istanbul Medipol University, Institute of Health Sciences, Department of Physiotherapy and Rehabilitation, Istanbul, Turkey

^6^ Department of Biophysics, School of Medicine, Istanbul Medipol University, Istanbul, Turkey

e-mail: [bguntekin@medipol.edu.tr](mailto:bguntekin@medipol.edu.tr)

**Cognitive Neurodynamics**

**Application of Visuospatial Attention Paradigm**

The images for the paradigm were created using the Adobe Photoshop software application. A total of 60 congruent and 60 incongruent stimuli were included in the paradigm. The 120 images in the paradigm appeared randomly on the screen during the testing of each participant. Each stimulus remained on the screen for 2 seconds (s). The subjects were initially asked to focus on the plus sign located at the center of the screen. The subjects were asked to find the target stimulus inside the circles during this process. Later, when the 1 s response screen appeared, the subjects were asked to press 1 if the target shape inside the circles was a ‘square’ and 3 if it was a ‘diamond’. When the response screen ended, the ISI screen appeared randomly between 2-3 s. The transition between the response screen and the ISI screen was not noticeable. The responses given on the ISI screen were not accepted. A trial application of 8 stimuli was performed before the actual testing. The paradigm flow diagram is shown in Figure 3a.

**Application of Mental Rotation Paradigm**

Each stimulus will remain on the screen for five seconds. During this time, the subjects were asked to find out whether the shape on the right was the same as the shape on the left. A total of 90 different stimuli were used (60 congruent stimuli, 30 incongruent stimuli). At the end of the 5 s MR process, a 1 s response screen was added to the paradigm, followed by a randomized 2-3 s ISI screen. Subjects were asked to press 1 if the two shapes were the same, and 3 if they were different. The transition between the response screen and the ISI screen was imperceptible. Responses given on the ISI screen were not accepted. A training with 8 stimuli was applied before the actual testing. The paradigm's flow diagram is shown in Figure 3b.

Exercise program

| Application Weeks | Applied Exercises |
| --- | --- |
| 1. and 2. weeks exercises | On a flat, hard surface;  -Weight bearing in 4 directions on 2 feet without taking a step  -Taking steps forward and backward (separately for each foot)  -Taking a tandem position and maintaining the position (separately for each foot)  -Standing on one foot (separately for each foot)  -Weight bearing in 4 directions by taking step  -Taking a step back towards the step  -Taking a step onto the balance board |
| 3. and 4. weeks exercises | On a flexible-soft surface (mat);  -Weight bearing in 4 directions on 2 feet without taking a step  -Take steps forward and backward (separately for each foot)  -Take a tandem position and maintain the position (separately for each foot)  -Stand on one foot (separately for each foot)  -Weight bearing in four directions by taking step  -Take a step back towards the step  -Take a step onto the balance ball |
| 5. and 6. weeks exercises | On the balance board;  -Weight bearing in 4 directions on 2 feet without taking a step  -Take steps forward and back (separately for each foot)  -Go into tandem position and maintain position (separately for each foot)  -Stand on one foot (separately for each foot)  -Weight bearing to 4 directions by taking step  -Catching balls thrown by the practitioner |

Video-based Balance Games Program

| Application Weeks | Applied Games |
| --- | --- |
| 1. week | - Basic Step  - Penguin slide (weight bearing to the left and right)  - Tightrope Tension (weight bearing to the left and right) (basic version)  - Heading (weight bearing to the left and right) (basic version) |
| 2. week | - Basic Step  - Penguin slide (weight bearing to the left and right)  - Tightrope Tension (weight bearing to the left and right) (basic version)  - Heading (weight bearing to the left and right) (basic version) |
| 3. week | - Basic Step  - Penguin slide (weight bearing to the left and right)  - Heading (weight bearing to the left and right) (basic version)  - Table tilt (weight bearing to all directions) (basic version) |
| 4.week | - Advanced Step  - Penguin slide (weight bearing to the left and right)  - Heading (weight bearing to the left and right) (advance version)  - Table tilt (weight bearing to all directions) (basic version) |
| 5.week | - Advanced Step  - Table tilt (weight bearing to all directions) (advance version)  - Balance bubble (weight bearing to all directions) (basic version)  -Ski slalom (weight bearing to all directions) (basic version) |
| 6.week | - Advanced Step  - Table tilt (weight bearing to all directions) (advance version)  - Balance bubble (weight bearing to all directions) (advance version)  - Ski slalom (weight bearing to all directions) (advance version) |

Features and aims of games

| **Games** | **Features of Games** |
| --- | --- |
| **Step Basic** | People need to take appropriate steps onto the balance board in coordination with the warnings on the screen. It includes steps forward, backward and sideways. |
| **Penguin Slide** | While on the balance board, the players need to direct the penguin they see on the screen left and right on an iceberg and collect the fish that come to them. |
| **Tightrope Tension** | While on the balance board, people must maintain their balance on a rope and move towards the finish line by transferring weight left and right. |
| **Heading** | While on the balance board, the players are required to catch balls thrown at them from all directions by transferring their weight in the goalkeeper position. In the meantime, they are required to avoid non-target stimuli (such as shoes). |
| **Table Tilt** | While on the electronic balance board, people need to direct the balls they see on the screen to the appropriate spaces by transferring weight in all directions. |
| **Ski Slalom** | While the players are on the balance board, they must transfer their weight in all directions and get the skier on the screen past the flags, which are the target stimuli, to the finish line. |
| **Advance Step** | People need to take appropriate steps on the balance board in coordination with the warnings on the screen. Unlike the Basic Step game, it includes rotational movements. |
| **Balance Bubble** | People must bring the character they see on the screen to the finish line over a river by transferring weight in all directions, without hitting the surrounding rocks and staying away from non-target stimuli (such as bees). |
